# Delayed Induction of Human NTE (*PNPLA6*) Rescues Neurodegeneration and Mobility Defects of *Drosophila swiss cheese (sws)* Mutants

**DOI:** 10.1371/journal.pone.0145356

**Published:** 2015-12-15

**Authors:** Alyson Sujkowski, Shirley Rainier, John K. Fink, Robert J. Wessells

**Affiliations:** 1 Department of Physiology, Wayne State University School of Medicine, Detroit, Michigan, United States of America; 2 Department of Neurology, University of Michigan Medical School, Ann Arbor, Michigan, United States of America; 3 Geriatric Research Education and Clinical Center, Ann Arbor Veterans Affairs Medical Center, Ann Arbor, Michigan, United States of America; Alexander Fleming Biomedical Sciences Research Center, GREECE

## Abstract

Human *PNPLA6* gene encodes Neuropathy Target Esterase protein (NTE). PNPLA6 gene mutations cause hereditary spastic paraplegia (SPG39 HSP), Gordon-Holmes syndrome, Boucher-Neuhäuser syndromes, Laurence-Moon syndrome, and Oliver-McFarlane syndrome. Mutations in the *Drosophila* NTE homolog *swiss cheese (sws)* cause early-onset, progressive behavioral defects and neurodegeneration characterized by vacuole formation. We investigated *sws* ^*5*^ flies and show for the first time that this allele causes progressive vacuolar formation in the brain and progressive deterioration of negative geotaxis speed and endurance. We demonstrate that inducible, neuron-specific expression of full-length human wildtype NTE reduces vacuole formation and substantially rescues mobility. Indeed, neuron-specific expression of wildtype human NTE is capable of rescuing mobility defects after 10 days of adult life at 29°C, when significant degeneration has already occurred, and significantly extends longevity of mutants at 25°C. These results raise the exciting possibility that late induction of NTE function may reduce or ameliorate neurodegeneration in humans even after symptoms begin. In addition, these results highlight the utility of negative geotaxis endurance as a new assay for longitudinal tracking of degenerative phenotypes in *Drosophila*.

## Introduction

Mutations in human NTE (also known as patatin-like phospholipase domain containing protein 6, PNPLA6) cause diverse human neurologic disorders including hereditary spastic paraplegia (HSP), Gordon-Holmes syndrome, Boucher-Neuhäuser syndrome, Laurence-Moon syndrome, Oliver-McFarlane syndrome, and Leber’s congenital amarosis [1–3]. NTE has lysophospholipase B activity, converting lysophosphatidylcholine to glycerophosphocholine [4]. Current animal models for PNPLA6-associated pathologies include the adult hen and mouse, but high cost and variable phenotypic presentation create significant limitations to these systems [5]. Previous studies have established precedents for the use of mouse, hen, and recombinant human enzyme both in vitro and in vivo [5, 6]. Deletion of the mouse homolog is lethal [7], while brain-specific knockout mice show progressive neurodegenerative phenotypes [8].

The *Drosophila* homologue (sws) of human NTE appears to regulate cAMP-dependent protein kinase. [9, 10]. Null *sws* ^*1*^ mutants reach adulthood but have a reduced lifespan and exhibit progressive neurodegeneration [11]. Neurodegeneration in these animals involves neuronal and glial cell death in the brain, leading to the appearance of characteristic vacuoles [12] that can be rescued by continuous transgenic expression of either the fly, mouse or human homolog of NTE [12, 13].

*sws* ^*5*^ is an EMS-generated point mutant [11] causing a glycine to arginine change in a highly-conserved residue near the reported PKA binding region [9]. *sws* ^*5*^ flies have shortened lifespans similar to or more severe than the null mutant [11]. We examined the ability of wildtype human NTE to rescue neurodegeneration and mobility defects of *sws* ^*5*^ mutants.

For the first time, we assessed whether post-developmental induction of a rescue construct could still ameliorate the neurodegenerative symptoms of a *sws* mutant. To provide temporal control of transgene induction, we used the RU-inducible gene switch system to induce neuron-specific expression of hNTE in adult *sws* mutant flies. For this, full-length hNTE was cloned into pUAST for use in the UAS/Gal4 expression system and a stable, mifepristone-inducible, pan-neuronal expression line (GS-elav-Gal4>UAS-hNTE) was crossed to *sws* ^*5*^ mutant females. Male progeny receiving RU486 (RU+) after reaching adulthood expressed hNTE pan-neuronally in a hemizygous *sws* ^*5*^ background and are referred to as “hNTE rescue” flies. Flies not receiving RU486 (RU-) are genetically identical siblings that received only vehicle, thus expressing the hemizygous mutant phenotype, but in a background identical to the rescue flies. These are used as a direct comparison for rescue, due to their identical genetic background with the rescue flies, distinct from the wild-type Berlin K background or the *sws* ^*5*^ mutant in the Berlin K background.

It has been previously observed that neurodegeneration causes impaired mobility in *Drosophila* [14–16]. Furthermore, both induced locomotor activity and endurance have been shown to undergo a characteristic age-related decline in wild type flies [17–19]. In addition to standard neurodegeneration assays, we utilized an automated negative geotaxis machine, known as the Power Tower [19, 20], to monitor endurance as a novel assay for neurodegeneration in *Drosophila*.

## Results

### hNTE Rescues *sws* ^*5*^ Esterase Activity

**Fig 1:**
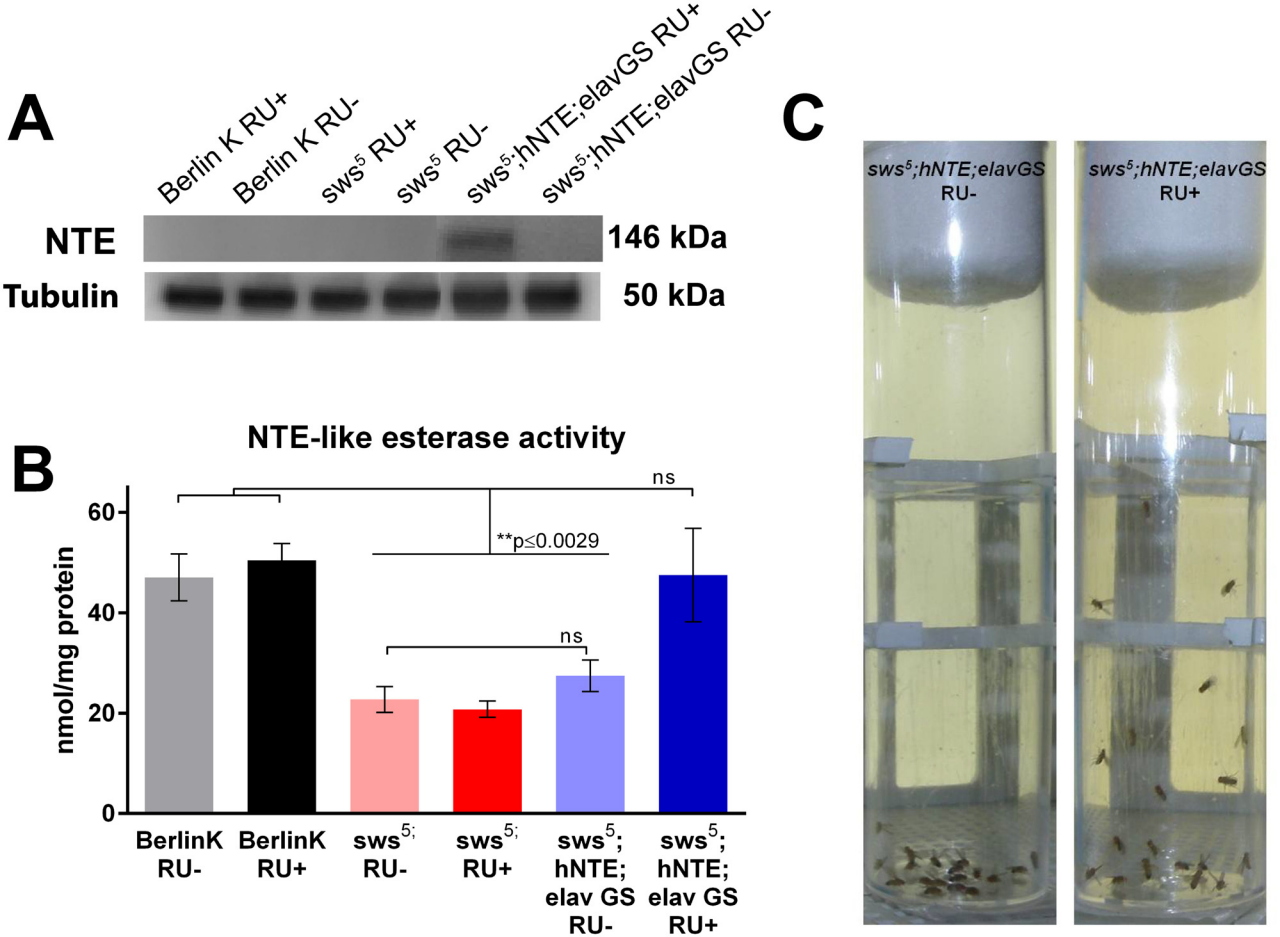
Adult-specific hNTE expression restores NTE-like esterase activity and improves negative geotaxis in *sws* ^*5*^ deficient flies. (A) hNTE protein is present in heads of adult hNTE rescue flies, but not controls or *sws* ^*5*^ mutants. Loading control using anti-Tubulin is shown below. Representative image from 3 biological replicates. (n = 10 fly heads for each sample) (B) Esterase activity is reduced in *sws* ^*5*^ mutants and control RU- flies in comparison to wild-type Berlin K (pairwise t-test, p = 0.006 and p = 0.0177, respectively). Flies expressing hNTE using elav-GAL4 increase esterase activity to the level of the wild type Berlin K (pairwise t-test, p = 0.9668). Data across groups were analyzed using two-way ANOVA with Bonferroni post-test and represent duplicate biological samples (n = 10, p≤0.0029, genotype effect, p<0.0001, RU effect, p = 0.0094, RU-by-genotype effect, p = 0.0038). (C) Age-matched *sws* ^*5*^ *;hNTE;elav GS* RU *+* flies display increased climbing vigor in comparison to flies without hNTE induction. Mifepristone (RU+) or vehicle (RU-) was fed to adult flies 3 days post-eclosion and maintained throughout life in order to induce transgene expression after development was complete. Photograph was taken 2 seconds after sharp rapping of vials onto a solid surface in order to induce negative geotaxis response.

Virgin females homozygous for the *sws* ^*5*^ allele on the X chromosome were crossed to UAS-hNTE;elav GS males. Immediately following adult eclosion, transgene expression was induced by RU486 feeding and continued throughout adult life or until time of experimentation, whichever occurred first. Transgenic expression of hNTE in 10-day old *sws* ^5^ hemizygous male fly heads was confirmed by Western blot. hNTE was completely absent in Berlin K, *sws* ^*5*^ and control flies but highly expressed in hNTE rescue fly heads (Fig 1A). To confirm enzymatic activity of the transgene, we examined NTE-like esterase activity [21] in age-matched Berlin K, *sws* ^*5*^, hNTE rescue and control groups. *sws* ^*5*^ mutants and control transgenics had dramatically reduced NTE esterase activity, when compared to levels in the wild-type Berlin K line. Expression of hNTE completely restored NTE activity to the level of wild-type Berlin K flies (Fig 1B).

### hNTE Improves *sws* ^*5*^ Negative Geotaxis

**Fig 2:**
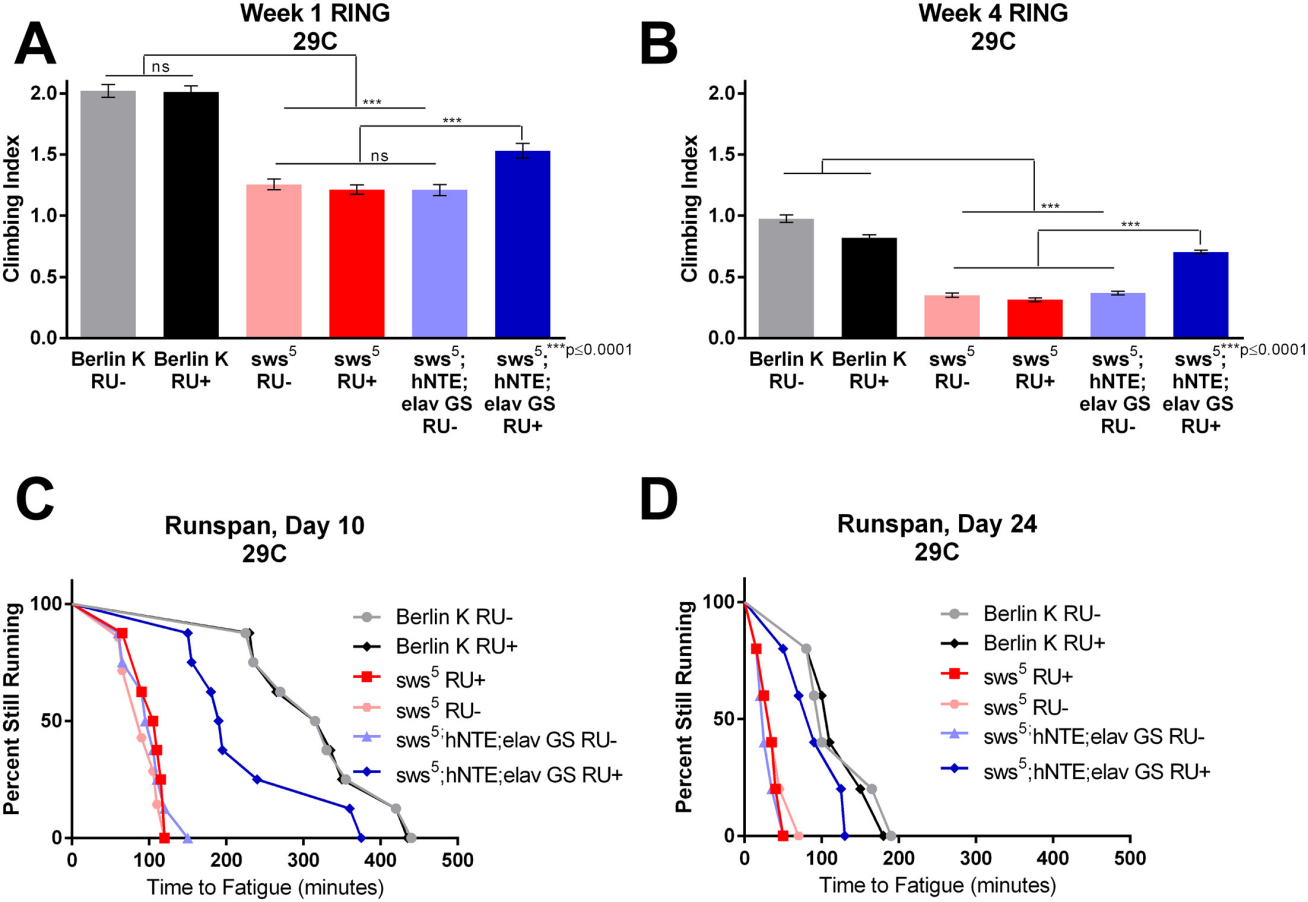
Continuous adult-specific hNTE expression improves climbing performance and endurance in *sws* ^*5*^ flies at 2 ages. (A) Average climbing ability is increased in hNTE rescue flies during week 1 of adult life in comparison to age-matched flies without hNTE expression (p≤0.0001), but not to the level of the wild-type Berlin K flies (p = 0.0001). Flies were assessed daily on days 4 through 8 at 29°C. Climbing performance was assessed on a minimum of 2 cohorts. Data were analyzed using two-way ANOVA with Bonferroni post-test. (n≥112, p≤0.0001, genotype effect, p<0.0001, RU effect, p = 0.0233, RU-by-genotype effect, p = .0003). (B)Average climbing performance assessed on days 25–29 (week 4) of adulthood is improved in hNTE rescue flies in comparison to age-matched flies without hNTE expression (p≤0.0001), but not fully rescued to wild-type levels (p≤0.0001). Climbing was assessed on a minimum of 2 separate biological cohorts. Data were analyzed using two-way ANOVA with Bonferroni post-test. (n≥84, p≤0.0001, genotype effect, p<0.0001, RU effect, p = .0043,RU-by-genotype effect, p<0.0001). Data from both timepoints (2A and 2B) were combined to assess effects across age, age effect, p<0.0001, RU effect, p<0.0001, interaction, p = 0.8656. Thus, there is an age-independent effect of RU on climbing. (C) Time to fatigue is increased in hNTE rescue flies compared to *sws* ^*5*^ flies and uninduced controls. Endurance experiments are analyzed statistically by log-rank (n≥150 p≤0.0001). Flies were aged at 29°C and assessed for time-to-fatigue on the 10^th^ day. (D) 24-day old control flies exhibit age-related decline in endurance relative to day 10. *sws* ^*5*^ mutants and RU- control flies display marked reduction in time-to-fatigue- in comparison to Berlin K flies (log-rank, p≤0.0001). Time-to-fatigue is increased in hNTE rescue flies compared to *sws* ^*5*^ and RU- controls (log rank, p≤0.0001), but not fully restored to wild-type (log rank with Pearson correlation, p = 0.0020). Endurance was assessed a minimum of 3 times to confirm reproducibility. n≥100 for all day-24 assessments.

Negative geotaxis, a measure of *Drosophila* climbing behavior, declines with age and neurologic impairment [17, 22]. Adult *sws* ^*1*^ mutant flies have shown early and progressive behavioral impairment [10]. *sws* ^*5*^ and RU- control flies also displayed severe mobility impairment in a stringent version of this assay measured over two seconds of climbing (Fig 1C). hNTE RU+ rescue flies had increased climbing performance compared to *sws* ^*5*^ mutants and to genetically identical RU- controls when assessed daily in weeks 1 and 4 of adulthood, although rescue did not reach the level of wild type Berlin K flies (Fig 2A and 2B). *sws* ^*5*^ cohorts were indistinguishable from RU- rescue flies (Fig 2A and 2B). All groups also showed significant decline with age from 1 to 4 weeks (Fig 2A and 2B).

### hNTE Improves *sws* ^*5*^ Endurance

We measured time to fatigue using the Power Tower, a machine that lifts and drops vials regularly to generate repetitive negative geotaxis stimuli [19]. Fig 2C and 2D plots the time-to-fatigue of each vial as a time-to-event curve, referred to as a “runspan” [18]. Vials are designated as fatigued when less than five flies out of 20 are climbing more than a centimeter up the tube after each drop. 10-day old Berlin K flies displayed typical endurance behavior, with the last vial of flies reaching fatigue after nearly 7 hours of continual climbing. In contrast, *sws* ^*5*^ mutant flies showed a severely accelerated fatigue. No vial of 10-day-old *sws* ^*5*^ mutant flies climbed >2 hours, a time-to-fatigue more typical of flies of five weeks of age or more [19]. Endurance in RU- rescue flies was not significantly different from *sws* ^*5*^ mutants. However, hNTE rescue cohorts displayed dramatically enhanced endurance. (Fig 2C). The improvement in time-to-fatigue in hNTE rescue flies was particularly impressive given that protein expression was not induced until adulthood.

To determine if rescue of endurance behavior persists with advancing age and neurodegeneration, time-to-fatigue was assessed in the same cohort of male flies on day 24 of adulthood. Both RU+ and RU- Berlin K flies showed an age-related decline in runspan. *sws* ^*5*^ flies also exhibited a severe reduction in endurance performance, with all vials reaching fatigue in just over an hour. Performance in RU- uninduced controls was indistinguishable from *sws* ^*5*^ mutants. Importantly, hNTE rescue flies fatigued at almost the same time as age-matched wild-types (Fig 2D), indicating that continued transgene expression provides long-term protection against accelerated neurodegeneration.

### Vacuolization of *sws* ^*5*^ Brains Is Reduced By hNTE

**Fig 3:**
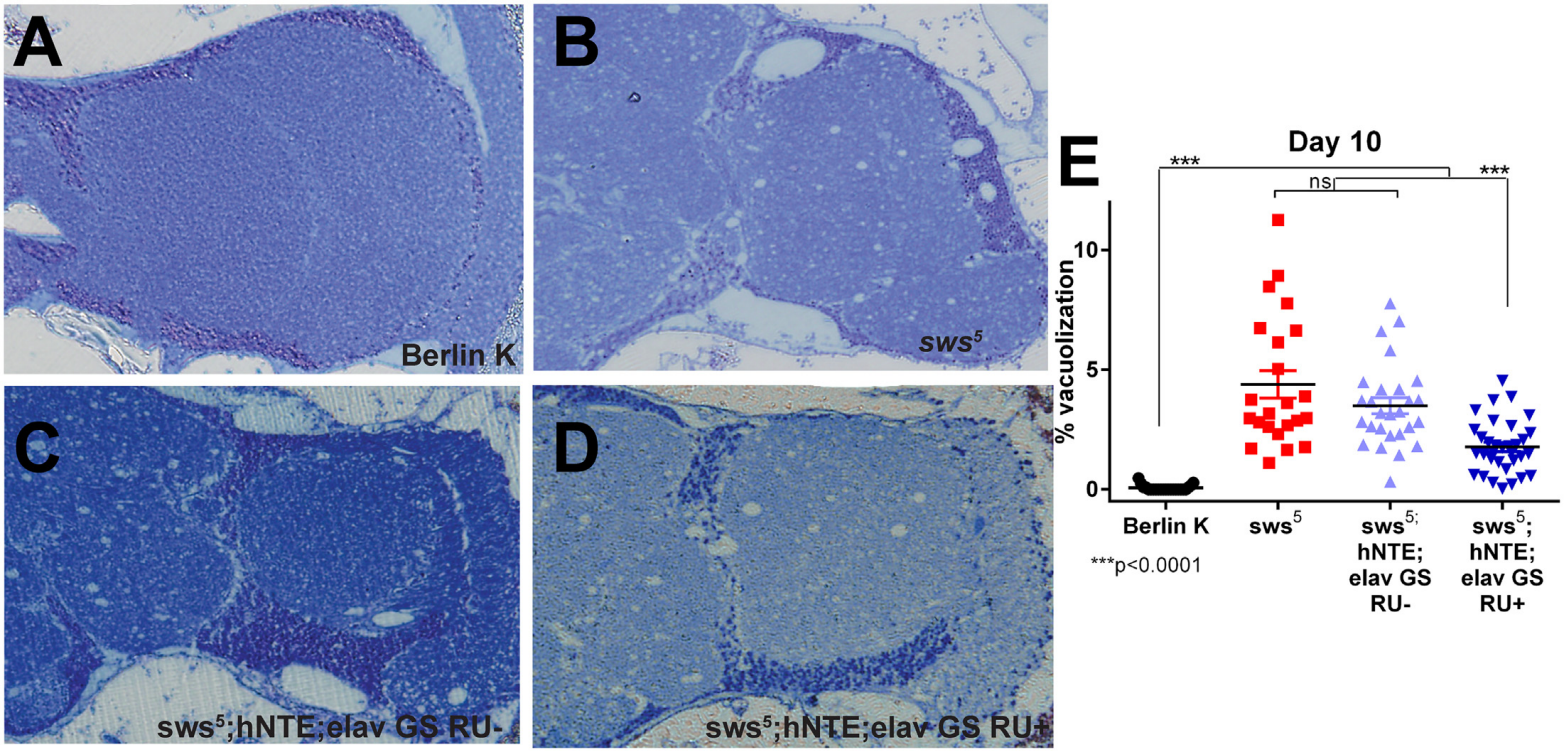
Adult-specific hNTE expression arrests vacuole expansion in *sws* ^*5*^ brains. Representative 40X images of 500 nm transverse sections (approximately 75μm caudal to first appearance of tissue) of *Drosophila* right hemisphere from 10-day old flies. Berlin K RU+/RU- and *sws* ^*5*^ RU+/RU- were morphologically identical, so a single image is presented for clarity. (A) Background control flies have no vacuoles present. (B) *sws* ^*5*^ and RU- control flies exhibit characteristic vacuolization. (C, D) *sws* ^*5*^ flies expressing adult-specific hNTE have reduced percent vacuolization when compared to mutants (as quantified in E). (E) Quantification of percent vacuolization phenotype from sectioned *Drosophila* brains. Berlin K RU+/RU- and *sws* ^*5*^ RU+/RU- cohorts did not display statistically significant differences in area of brain occupied by vacuoles and are pooled. Data represent total area of vacuoles divided by total sample area. Although overall RU effect is not significant, *sws* ^*5*^ flies expressing pan-neuronal hNTE display significantly less vacuolization area in comparison to uninduced mutants (pairwise t-test, p≤0.0001) and uninduced controls (pairwise t-test, p≤0.0001) (two-way ANOVA with Bonferroni post-test, p≤0.0001, genotype effect, p<0.0001, RU effect, p = 0.8707, RU-by-genotype effect, p = 0.9324). n≥23 flies of each genotype.

Widespread vacuolization caused by neuronal cell death has been previously observed in *sws* mutant fly brains [11, 23]. We evaluated the degree to which hNTE expression affected *sws* histopathology in age-matched cohorts 48 hours after endurance assessment. Wild-type Berlin K flies displayed normal brain morphology (Fig 3A). Brains from *sws* ^*5*^ flies displayed vacuoles throughout, as well as darkly stained areas characteristic of cell death and apoptosis (Fig 3B), as previously observed in brains from *sws* ^*1*^ and *sws* ^*4*^ flies [11].

RU- control flies displayed similar histology to *sws* ^*5*^ mutants. Multiple vacuoles of varying sizes were visible in all brain sections, and dark toluidine blue segments were apparent (Fig 3C). Although control and hNTE rescue flies had a similar number of vacuoles, measurement of average vacuole area per brain revealed less brain area lost to vacuolization (Fig 3D and 3E). Therefore, adult-specific neural hNTE expression appeared to reduce the rate of vacuole expansion without decreasing vacuole number.

### Delayed hNTE Expression Improves *sws* ^*5*^ Motor Function and Histopathology

As adult-induced rescue was able to improve mobility and reduce histopathology, we examined the effect of hNTE expression even later in adult life, after significant degeneration had occurred. We performed 4 consecutive endurance tests in conjunction with delayed induction of gene expression. Mutant, rescue, and control cohorts were allowed to eclose and mature at 29°C for one week. From day 7, all cohorts were provided either food containing RU for continuous hNTE induction (RU+) or food containing vehicle only (RU-).

**Fig 4:**
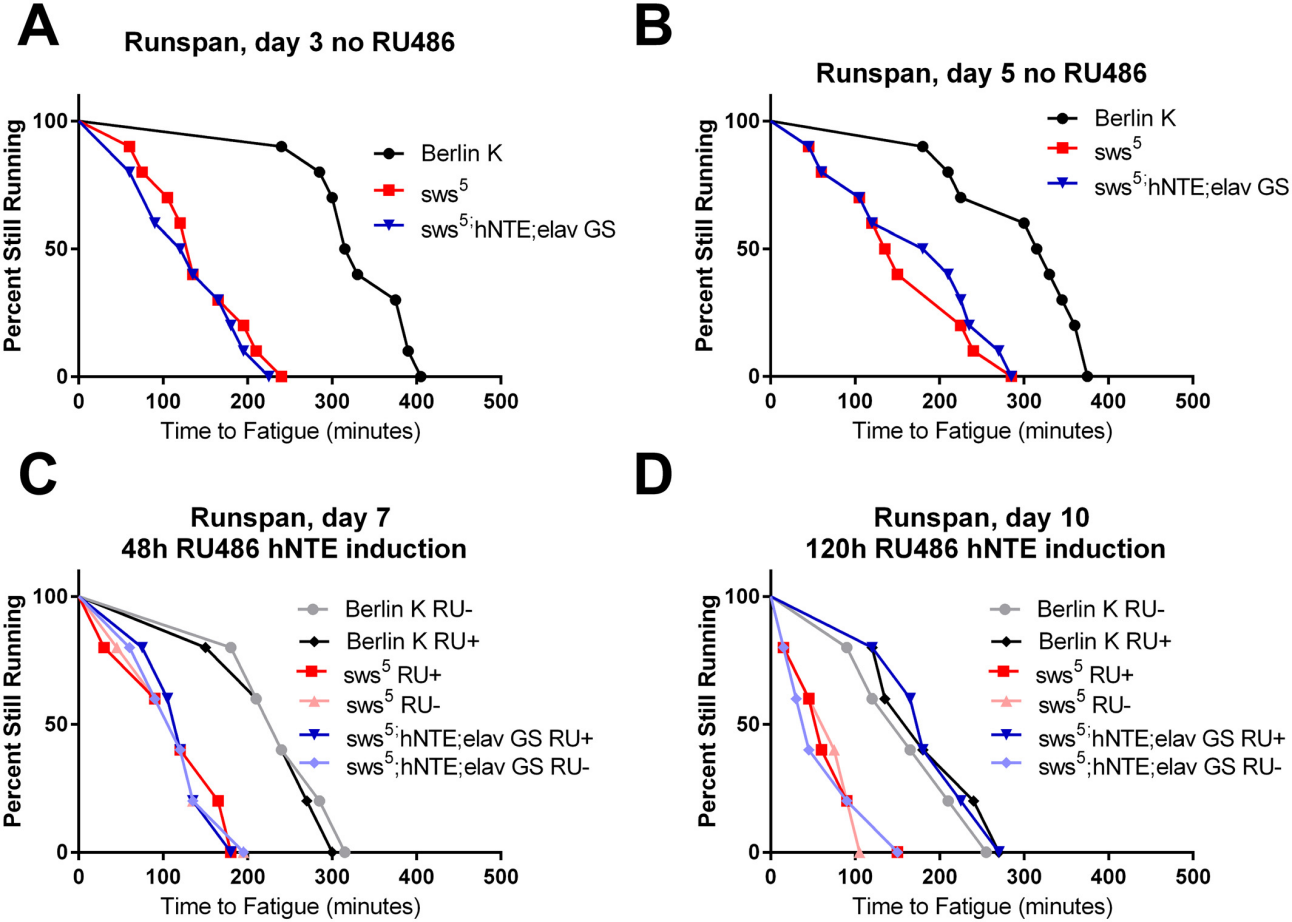
Delayed hNTE expression rescues endurance in *sws* ^*5*^ flies. Endurance tolerance was assessed before (A, B) and after (C, D) mifepristone (RU486) application and induction of hNTE expression. (A) Berlin K flies exhibit wild-type endurance on day 3 of adulthood, while both *sws* ^*5*^ mutant and *sws* ^*5*^ *;hNTE;elav GS* show a marked reduction in time-to-fatigue (n = 200 for all groups, log-rank, p<0.0001). (B) 48 hours later *sws* ^*5*^ mutant and *sws* ^*5*^ *;hNTE;elav GS* cohorts continue to exhibit much lower endurance than Berlin K controls (n = 200 for all groups, long-rank, p<0.0001). At the end of the day-5-test, cohorts are divided in half and split into induced (RU+) and control (RU-) groups. (C) The same flies from (A) and (B) are tested for endurance 48 hours after mifepristone induction. Berlin K flies begin to decline in performance compared to day 5, independent of RU486 feeding (log rank, p≤0.0001 for RU+ and RU-). *sws* ^*5*^ mutant and *sws* ^*5*^ *;hNTE;elav GS* cohorts show reduced endurance relative to Berlin K whether RU486-fed or not (n = 100 for all groups, long-rank, p<0.0001). (D) On day 10, after 120 hours RU486 induction, hNTE rescue flies’ (*sws* ^*5*^ *;hNTE;elav* GS RU+) time-to-fatigue is fully rescued to wild-type Berlin K endurance capacity (log rank, p = 0.9436). *sws* ^*5*^ mutant and RU- control groups remain deficient in time-to-fatigue (log rank, p<0.0001). n = 100 for all cohorts.

The first endurance assessment was performed three days after eclosion. Control Berlin K flies climbed continuously for a maximum time of 400 minutes. Both *sws* ^*5*^ mutant flies and RU- rescue flies showed severely impaired endurance in comparison to Berlin K (Fig 4A). On day five, time-to-fatigue was similar to day three, with wild-type flies displaying much better endurance than *sws* deficient flies (Fig 4B). Subtle shifts in maximal time to fatigue were observed in the 3 cohorts at this timepoint for reasons that are not clear. Importantly, the relative difference between control Berlin K and *sws* ^*5*^ deficient cohorts remained highly significant. Immediately after assessment on day five, flies were given either inducing RU486 or RU- vehicle, then reassessed two days later.

After seven days at 29°C, Berlin K flies began to exhibit a decline in endurance compared to day 5, as analyzed by log-rank (p≤0.0001) (Fig 4C). *sws* ^*5*^, control, and hNTE rescue cohorts all retain much lower time-to-fatigue than wild-type groups, and are not significantly different from one another (Fig 4C). Thus, hNTE rescue expression between days five and seven had little effect. *sws* ^*5*^ flies do not decline in endurance during the first week of age as much as wild-type flies do (p = 0.4072 and p = 0.2550 for ages 3–5, and 5–7 days), perhaps because their performance is so low already.

Time-to-fatigue on day ten was noticeably different from previous assessments. By this time, experimental flies had been exposed to the drug for 120 hours, allowing increased time for hNTE expression. Both *sws* ^*5*^ and RU- rescue flies continued to exhibit progressive decline in time-to-fatigue between day 7 and day 10 (log rank, p≤ 0.0001 for both) (Fig 4D). Surprisingly, hNTE rescue flies show enhanced endurance on day 10, with a time-to-fatigue fully rescued to the level of age-matched Berlin K flies (p = 0.9436) (Fig 4D). This result indicates that progressive decline in endurance of *sws* ^*5*^ mutants was fully reversed by 120 hours of hNTE expression. The effect of age on endurance across groups was highly significant for this experiment (chi-square 144.4, p<0.0001) and the RU treatment-by-age effect was also highly significant (chi-square 255.6, p<0.0001).

**Fig 5:**
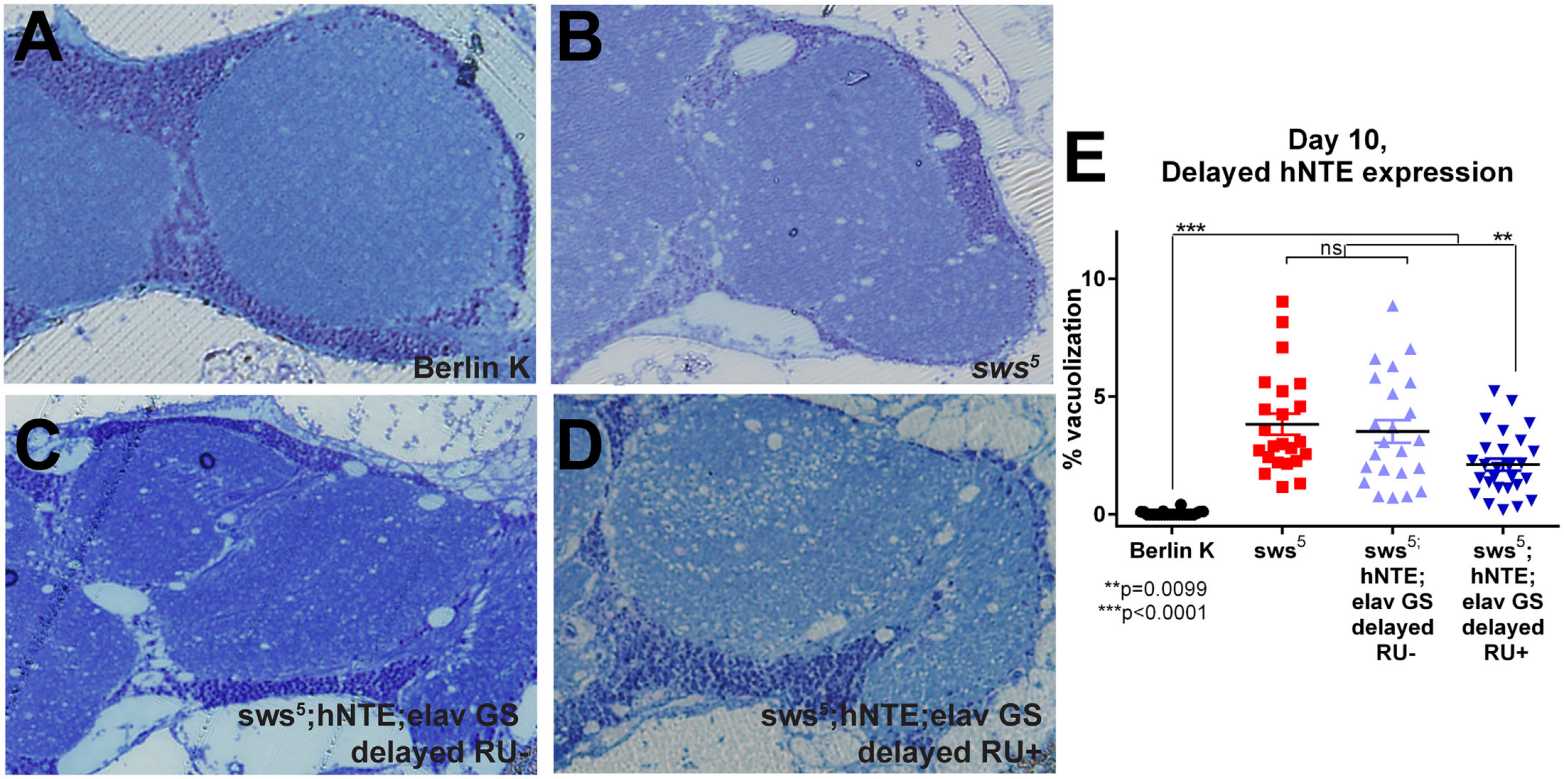
Delayed hNTE expression rescues vacuole size in *sws* ^*5*^ brains even after substantial disease progression. Representative 40X images of 500 nm horizontal sections of *Drosophila* brains from 10-day old flies with induced hNTE expression delayed until after one week of adulthood. Plane of sectioning, pooling of statistically and morphologically identical samples, and analyses are same as Fig 3. (A-C) Berlin K, *sws* ^*5*^ and control fly brains show similar histology to previously described cohorts. (D) Delayed hNTE expression rescues percent vacuolization to the same degree as expression throughout adulthood (p≤0.4405). (E) Quantification of percent vacuolization phenotype. Two-way ANOVA with Bonferroni post-test, pairwise p values as indicated in panel, overall genotype effect, p<0.0001, overall RU effect, p = 0.2236, overall genotype-by-RU effect, p = 0.0223. n≥23 flies of each genotype.

Following assessment of time-to-fatigue at 10 days, brains from flies of each cohort were sectioned within 48 hours in order to directly correlate brain histology with endurance. 10-day old Berlin K flies aged at 29°C showed wild-type morphology, as in Fig 3A. No vacuoles were present in any sample (Fig 5A). In our hands, brains of flies containing the *sws* ^*5*^ mutation appeared smaller than wild-type cohorts relative to the head capsule regardless of hNTE expression. Therefore, the plane of sectioning was measured in reference to the exterior of the head capsule for purposes of comparison (see Methods). *sws* ^*5*^ flies displayed vacuoles throughout all brain sections, many being very large in area (Fig 5B). Vacuoles in vehicle-fed rescue flies were also numerous and large in size (Fig 5C). Consistent with their improved fatigue tolerance, delayed hNTE rescue cohorts exhibited a lesser area of brain occupied by vacuoles than either *sws* ^*5*^ mutants or RU- rescue samples, although still more than wild-type controls (Fig 5D and 5E). This result further supports the idea that neurodegeneration as a result of reduced NTE activity is responsive to treatment even after substantial deterioration has occurred.

### hNTE Improves *sws* ^*5*^ Survival

**Fig 6:**
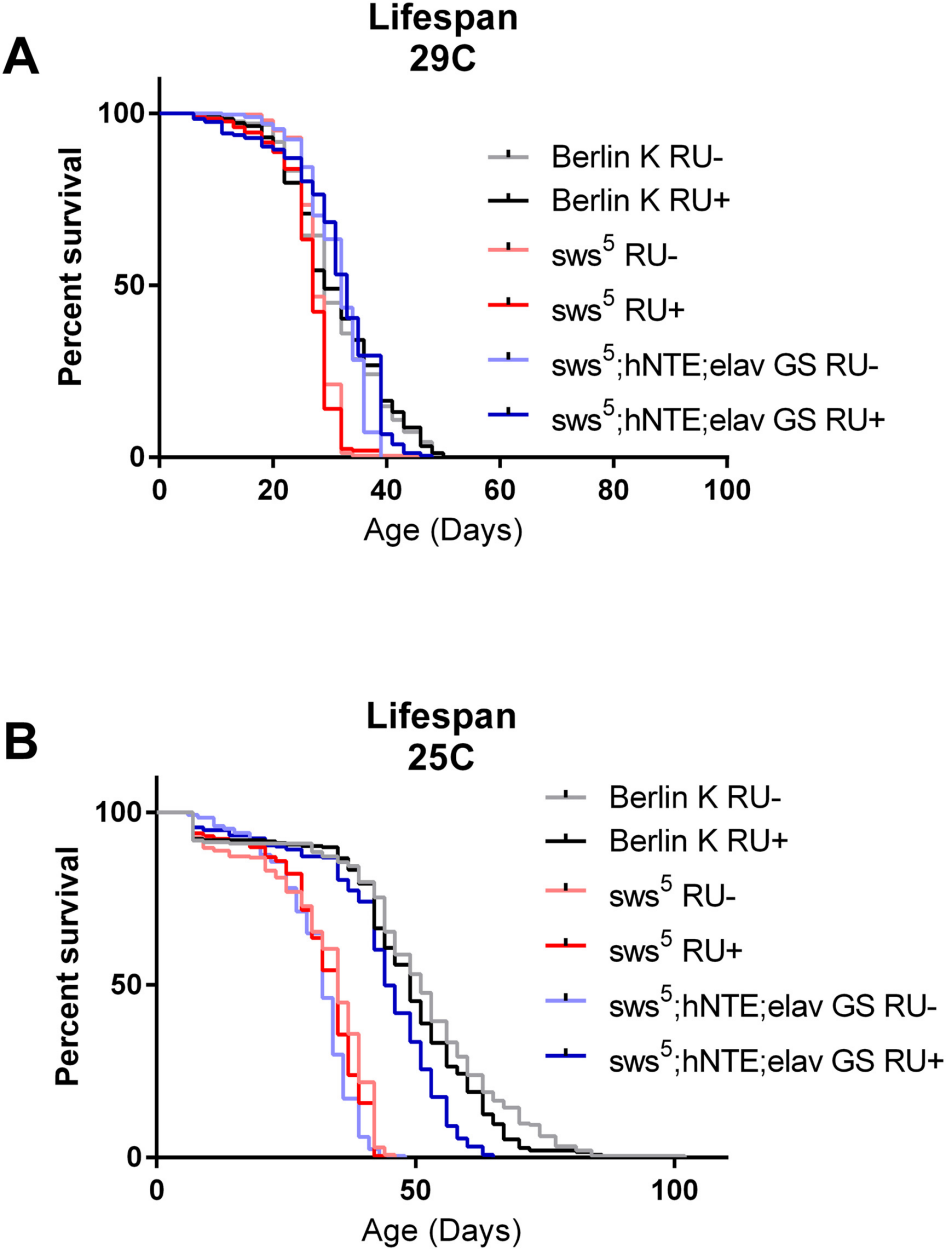
Adult-specific, pan-neuronal hNTE expression partially rescues reduced lifespan of *sws* ^*5*^ mutants at 25°C, 29°C. (A) *sws* ^*5*^ mutants have reduced lifespan at 29°C in comparison to both Berlin K RU+/RU- and *sws* ^*5*^ *;hNTE;elav GS* (RU+/RU-) flies (n≥248, log rank, p<0.0001). *sws* ^*5*^ *;hNTE;elav GS* RU- (RU- control) flies have slightly reduced lifespan relative to Berlin K RU+/RU- and *sws* ^*5*^ *;hNTE;elav GS* RU+ (hNTE rescue) flies (n≥248, log rank, p = 0.006) but are longer lived than *sws* ^*5*^ RU+/RU- flies (n≥248, log rank, p<0.0001). (B) *sws* ^*5*^ mutants (RU+/RU-) and RU- control flies have reduced lifespan at 25°C in comparison to Berlin K (RU+/RU-) and hNTE rescue flies (n≥243, log rank, p<0.0001). Survival of hNTE rescue flies is nearly fully rescued to wild-type Berlin K lifespan at 25°C but is still statistically significant (n≥247, log rank, p<0.0001.

Various *sws* mutants, including *sws* ^*5*^, have been previously shown to exhibit a reduction in lifespan [11]. Because adult-specific neural expression was sufficient to improve climbing ability, mobility, and brain histology, we examined its effect on survival in *sws* ^*5*^ flies. In our hands, *sws* ^*5*^ flies lived longer at 29°C than previously observed, with a maximum lifespan around 40 days (Fig 6A). Both median and maximal *sws* ^*5*^ lifespan was reduced, however, in comparison to Berlin K, RU- controls and hNTE RU+ rescue flies (Fig 6A). Since survival differences at 29°C were modest, we decided to repeat the experiment at a lower temperature, where wild type longevity is higher.

At 25°C *sws* ^*5*^ flies exposed to either RU486 or vehicle, and RU- control groups displayed marked reductions in both median and maximal lifespan relative to Berlin K control groups (Fig 6B). hNTE RU+ rescue flies exhibit a substantial rescue of survival at 25°C, with improvements in both median and maximum lifespan relative to mutant and RU- control groups (Fig 6B).

## Discussion

Neurodegenerative diseases are almost always diagnosed well after onset of symptoms [24, 25]. Reducing impairment after the development of disease is thus an important therapeutic goal. Significant neuronal cell death and vacuolization in *sws* ^*5*^ mutants is observed within the first ten days of life, while mobility impairments are apparent by day 3 of age.

Whereas previous studies also observed reduced vacuole formation in *sws* ^*1*^ flies overexpressing *Drosophila*, mouse, or human *PLNPA6* [12, 13], here we correlate reduced vacuole formation with longitudinal measures of mobility performance using a recently developed protocol for measuring *Drosophila* endurance. Measuring endurance with an automated system for inducing negative geotaxis behavior offers a non-invasive assessment of both coordination and strength, suitable for longitudinal assessment of performance decline. This system effectively measures performance decline as a result of either muscle-specific interventions (e.g. [26] or neuron-specific interventions (Sujkowski and Wessells, unpublished observation). This system can be useful for future assessments of degenerative disease models or as an assessment of functional aging.

Because hNTE expression was not induced in mutants until after development was complete, some developmental mobility defects could have persisted in adult hNTE rescue flies. However, hNTE rescue flies exhibited consistently faster climbing speed and higher endurance in comparison to both *sws* ^*5*^ mutants and transgenic controls. Furthermore, induction of rescue even after substantial decline has taken place in adults can not only slow decline, but actually reverse the decline in endurance of a longitudinally measured cohort.

Although we demonstrate reversal of mobility decline by late rescue, we do not find a reversal of vacuole formation. Rather we find that rescue strongly decreases the percent vacuolization at later time points. This result is consistent with the idea that rescue slows the expansion of existing vacuoles.

Taken together, these observations imply that protection of mobility may not absolutely require reversal of vacuole formation. Blocking the formation of additional vacuoles, or blocking the expansion of existing vacuoles may be sufficient to allow some degree of functional recovery. These results support the idea that induction of NTE activity may be therapeutically valuable even after substantial degeneration has occurred. Extending these studies to vertebrate models of NTE mutation will be critical.

## Materials and Methods

### Creating transgenic *Drosophila* lines

*hNTE* cDNA was generously provided by Paul Glynn. The cDNA sequence was ligated into the p[PUAST] expression vector for use in the UAS/Gal4 ectopic expression system. Transgenic flies were created by injecting p[PUAST]-hNTE constructs into *w* ^*1118*^ Drosophila embryos. A stable line containing both UAS-hNTE and a drug-inducible GS-elav-Gal4 was generated using standard genetic cross schemes.

### Fly Stocks and Maintenance

All parental lines were reared at 25°C, 50% humidity with a 12 hour light-dark cycle and provided with a standard 10% yeast/10% sucrose diet. *w* ^*1118*^ and Berlin K were obtained from the Bloomington Drosophila Stock Center; *sws* ^*5*^ flies (generated in a wild-type Berlin K background [11]) were generously provided by Dr. Doris Kretzschmar. GS-elav-Gal4 was generously provided by Scott Pletcher (University of Michigan), then mobilized to the third chromosome and backcrossed for 10 generations into *w* ^*1118*^. Resulting rescue flies were subsequently in a mixed *w* ^*1118*^-Berlin K background. Possible genetic background effects were further controlled by using GS-elav-Gal4 and defining RU- flies of the same mixed background as the negative control.

Experimental F1 male progeny were reared and aged as follows: 20 *sws* ^*5*^ virgin females and 5 UAS-hNTE;elav GS males were mated in 300 mL bottles with 50 mL standard 10% sucrose/10% yeast diet at 25°C, whereupon eggs were allowed to undergo and complete development. Adult progeny were age-matched by collecting within 2 hours of eclosion over a 48 hour time period. At least 400 adult males were collected for each genotype and immediately transferred into narrow polypropylene vials containing 5mL standard 10% yeast/ 10% sucrose food medium. Populations were split into “induced” and “control” flies and transferred to 5mL standard food medium containing either 100 μM mifepristone or 70% ethanol vehicle, respectively. Experimental and control flies were then housed at 29°C and allowed to age on either RU486 or vehicle until experimentation.

For delayed-induction experiments, synchronous male progeny were allowed to complete development at 25°C as above, and then aged for an additional 72 hours at 29°C on standard 10% yeast/10% sucrose media prior to being split into “induced” and “control” groups. Aged flies were then transferred to 5mL vials containing the addition of mifepristone or vehicle. All experimental groups were transferred to appropriate fresh food every second day. Survival analyses were performed concurrently on days of transfer.

### Western Blots

50 flies of each genotype were decapitated and separated. Triplicate biological samples of 10 isolated heads were homogenized and equal amounts of protein were loaded per lane. Gels and blots were performed as previously described [27]. Mouse monoclonal anti-hNTE (generously provided by Dr. Rudy Richardson) was used at 1:500. Following hNTE detection, blots were stripped and re-probed with mouse anti-tubulin (mouse monoclonal T5168, 1:10,000; Sigma-Aldrich) as loading control. Anti-mouse secondary antibody (Jackson Immunoresearch) was diluted to 1:5000.

### Esterase Assays

NTE was inhibited by preincubation with organophosphorus (OP) compounds as in Makhaeva et al. [6]. Homogenates from isolated *Drosophila* heads were diluted in 50 mM Tris-HCl, 1 mM EDTA, pH 8.0 at 37°C. NTE activity was defined as the Phenyl valerate (PV) hydrolase activity inhibited by preincubation for 20 min with 40 μM paraoxon (PO) but not abolished by preincubation for 20 min with 40 μM PO plus 50 μM mipafox (MIP) [5, 28]. 50 decapitated heads were assessed for each genotype, and duplicate biological samples were tested. Total protein from isolated heads was assessed in parallel using the Pierce BCA Protein Assay Kit (Rockford, IL).

### Negative Geotaxis Behavior

Flies were transferred into fresh polypropylene vials in a RING apparatus as in [17, 19] and allowed to equilibrate. Negative geotaxis was induced by rapping the fly vials 4 times in rapid succession. Fly positions were captured as digital images 2 seconds after eliciting climbing behavior. ImageJ analysis software [29] computed the distance climbed by each fly (NIH, Bethesda, MD). Distances were converted into quadrants using Microsoft Excel. The performance of at least 100 flies (five to six vials of 20 flies each) was calculated as the average of four consecutive trials to determine the average distance climbed per vial. Data were collected daily for 5 days in weeks 1 and 4. Daily measurements during each week were averaged across the five days and presented as average weekly climbing speed. Between RING assessments, flies were returned to appropriate media and returned to 29°C until the following day. Weekly data were analyzed using two-way ANOVA with Bonferroni post-test to compare significantly different values. Negative geotaxis experiments were repeated on a minimum of 2 biological cohorts.

### Endurance

Similar to negative geotaxis behavior, endurance in *Drosophila* shows a characteristic age-related decline. [18, 19]. Flies are divided into 8 groups of 20 individuals and placed on an automated negative geotaxis inducing machine [19, 20]. When fewer than 5 flies in a vial are able to climb above 1 cm, the vial is scored as “fatigued” and removed from the apparatus.

After 10 days of aging at 29°C, 5 to10 vials of flies from each genotype were assessed for time-to-fatigue using the endurance assay described by Tinkerhess et al. [19]. Each data point represents the time at which a vial of flies was removed and scored as “fatigued”. Data were presented as survival curves and analyzed by log rank. At 24 days of aging at 29°C, approximately 20% of the flies from each cohort had died, but survival proportions for all groups was not significantly different when analyzed by log rank. For delayed-induction experiments, 10 vials of each cohort were assessed on normal 10% sucrose/10% yeast diet at days 3 and 5. At the conclusion of the day-5 endurance test, the same 10 vials were split into “induced” and “control” groups and placed on RU486 food, or normal diet containing an equal amount of 70% ethanol vehicle, respectively. Thus, 5 vials of 20 flies were assessed for fatigue tolerance in each cohort. Runspan graphs containing less than the previously-stated number of data points indicate that more than one vial reached fatigue at the same time. All endurance experiments were repeated a minimum of 3 times to confirm reproducibility.

### Adult Brain Histology

Live flies were fixed in 4% paraformaldehyde, 2.5% glutaraldehyde as previously described [11]. Transverse sections of approximate midbrain were obtained by taking 150 500 nm slices from the first appearance of tissue and mounting the slice at the final plane. Light microscopy was performed on 500 nM-thick horizontal sections stained with 1% toluidine blue and 1% borax in water. Bright field microscopy was performed using an Olympus BX-51 upright light microscope outfitted with an Olympus DP-70 camera and software. Both central brain and optic lobe were analyzed for appearance of vacuoles by both number and % area using ImageJ (Bethesda, MD) software. Data were exported to Microsoft Excel for quantification. Vacuolization of brain tissue was not found to be significantly different between brain regions analyzed (optic lobe and central brain) and were subsequently pooled. Vacuolar area was not found to be significantly different between Berlin K RU- and RU+ nor between *sws* ^*5*^ RU- and RU+ and were similarly pooled for analysis.
